# Monomer-Shuffling and Allosteric Transition in KaiC Circadian Oscillation

**DOI:** 10.1371/journal.pone.0000408

**Published:** 2007-05-02

**Authors:** Mitsumasa Yoda, Kohei Eguchi, Tomoki P. Terada, Masaki Sasai

**Affiliations:** 1 Department of Computational Science and Engineering, Nagoya University, Nagoya, Japan; 2 CREST-Japan Science and Technology Agency, Nagoya, Japan; University of Glasgow, United Kingdom

## Abstract

Circadian rhythms in living organisms have long been attributed solely to a transcription-translation loop comprising a negative or positive feedback. The rhythms in cyanobacteria are known to be modulated by *kaiC, kaiA* and *kaiB* genes. It was recently shown, however, that their product proteins KaiC, KaiA and KaiB are sufficient to reconstitute the circadian rhythm in the phosphorylation level of KaiC *in vitro*. It has since been unclear why such an oscillatory behavior can occur in the absence of the apparent transcription-translation feedback. In the meantime, it has been reported that the monomer exchange between KaiC hexamers occurs in a phosphorylation-dependent manner, which suggests that the monomer shuffling is also involved in the circadian rhythm (H. Kageyama *et al., Mol. Cell*, **23**, 161 (2006)). To further clarify the role of the monomer shuffling, we have performed a computational modeling of interactions among Kai proteins assuming the allosteric transition of KaiC hexamer as well as the monomer shuffling. The results show that the existence of both monomer shuffling and allosteric transition can synchronize the phosphorylation level of the KaiC hexamers, and stabilizes its oscillation.

## Introduction

Biological activities of diverse organisms have circadian rhythms to fit to the day/night cycle of the environment [1]. The simplest organisms known to show circadian rhythms are cyanobacteria [2]. Three genes, *kaiA, kaiB*, and *kaiC*, play essential roles in the circadian clockwork of a cyanobacterium, *Synechococcus elongatus* PCC 7942 [3], in which the expressed protein, KaiC, represses the expression of *kaiB* and *kaiC* and thus constitutes a negative feedback loop [3], [4]. Various feedback regulations among clock genes were found in many other organisms, which led to a view that the transcription-translation oscillation is the source of circadian rhythmicity [5]. The generality of this view, however, is now being questioned at least for cyanobacteria [6]. Recent report of Nakajima *et al*. showed that the temperature-compensated robust circadian oscillation of KaiC phosphorylation can be reconstituted by incubating three Kai proteins, KaiA, KaiB, and KaiC, with ATP *in vitro* [7]. In this experiment, the reaction mixture contained neither mRNA nor DNA and the amount of protein molecules remained constant, which indicates that not the transcriptional regulation but Kai protein complexes are the pacemaker of cyanobacterial circadian clock [8].

As there is no transcription-translation feedback loop in this system, it is not clear at present why the number of phosphorylated KaiC can exhibit such a stable oscillation pattern. There have been a few preceding studies using theoretical models aiming at elucidating how this protein-only system can generate the robust circadian oscillation. With the limited experimental information, however, the theoretical model builders were forced to make some basic assumptions at their starting point [9]–[11]. Emberly and Wingreen assumed that KaiC monomers are shuffled during the period of phosphorylation of KaiC and that KaiC hexamers aggregate to form mesoscopic clusters during the period of KaiC dephosphorylation [9]. Kurosawa *et al*. assumed that both KaiA and KaiB have multiple states and regulate the KaiC phosphorylation process in multiple ways [10]. Mehra *et al*. assumed that the phosphorylated KaiC molecule catalyzes phosphorylation of other KaiC molecules [11]. Takigawa-Imamura et al. assumed that there are two sequential states of phosphorylated KaiC and that KaiA can bind to unphosphorylated KaiC and the second, but not to the first, state of phosphorylated KaiC [12]. Miyoshi *et al.* assumed that KaiB have two states which can be converted to each other with the help of KaiC hexamers at the different levels of phosphorylation [13]. These assumptions have not yet been confirmed experimentally and the consensus is not reached at the moment.

More recently, Kageyama *et al*. examined the detailed features of KaiC oscillation *in vitro* [14]. Their experimental data provided a new opportunity for testing the validity of theoretical models. Shuffling of monomers, for example, was shown to be most frequent during the dephosphorylation period [14], showing that the scenario of Emberly and Wingreen does not hold in its original form. In this paper, we exploit the data of Ref.14 to propose a consistent model of Kai protein complex dynamics. Our model presented here conforms to these experimental findings as well as other known structural and physicochemical information.

KaiA catalyzes the phosphorylation of KaiC [15] and KaiB attenuates the kinase activity of KaiA [16], where KaiA interacts with KaiC mostly in the KaiC phosphorylation phase and the KaiB activity is most evident in the KaiC dephosphorylation phase [14], [17]. We assume that the difference in binding affinities toward KaiA and KaiB can be attributed to the presence of two structural states of KaiC hexamer: In the phosphorylation phase KaiC has higher binding affinity to KaiA than to KaiB and in the dephosphorylation phase KaiC has higher binding affinity to KaiB than to KaiA. Such assumption of the concerted conformational change of monomers within homooligomers is well known to successfully describe the cooperativity in the ligand binding of hemoglobin [18] and chaperonin [19], both of which are correlated to changes in their quaternary structures [20], [21].

Another key assumption in our model is on the shuffling of KaiC monomers. KaiC is known to form a hexamer both in solution and crystals [22], [23], [14]. We assume that KaiC monomers are quickly interchanged when two hexamers interact, which is based on the experimental result [14] that the shuffling proceeds without accumulation of smaller or greater species than hexameric KaiC. The fact that the shuffling is most frequent in the dephosphorylation phase [14] indicates that intermonomer interactions are destabilized in the structural state in the dephosphorylation phase compared to that in the phosphorylation phase. Therefore, we denote the structural state which dominates in the dephosphorylation phase by relaxed (R) state, whereas the structural state which dominates in the phosphorylation phase is denoted by tense (T) state.

**Figure 1:**
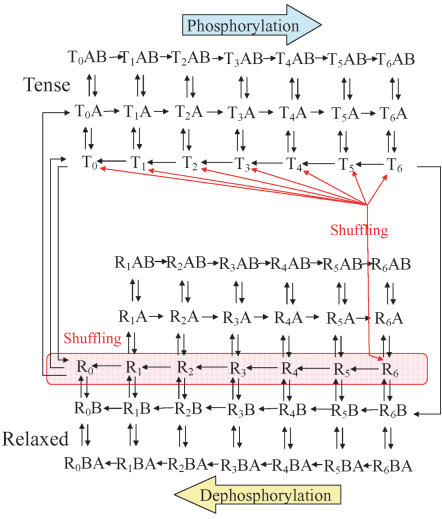
Reaction scheme assumed in the model. Association/dissociation, kinase/phosphatase reactions, and allosteric transitions are indicated by black arrows. Monomers of KaiC can be shuffled between T and R_6_ (red arrows) and between two Rs (pink area).

Each KaiC monomer has two phosphorylation sites at Ser431 and Thr432 [24]. Although the close distance (7.4Å) between these two sites in crystal structure [25] indicates that phosphorylation at these two sites should not be independent, the detailed mechanism of the correlation between the two sites is unknown at present. For simplicity, we do not distinguish two sites explicitly and assume that phosphorylation on one of the two sites is essential for the circadian oscillation in the present paper. When we also neglect the difference in the location of phosphorylated monomer within the hexamer, phosphorylation states of KaiC hexamer is specified by the total number of phosphorylated KaiC monomers within hexamer. Therefore there are 14 distinct states for KaiC hexamer denoted by T*_i_* or R*_i_*, where T or R denotes the structural state and *i* is the number of phosphorylated monomers from 0 to 6. Analyses of thus simplified model should provide a starting point to clarify the essential mechanism of the circadian oscillation produced by dynamically interacting proteins. The reaction scheme assumed in our model is shown in Fig. 1, where KaiA and KaiB are denoted by A and B, respectively. We consider Kai protein complexes, KaiC-KaiA (denoted by T*_i_*A or R*_i_*A), KaiC-KaiB (R*_i_*B), KaiC-KaiA-KaiB (T*_i_*AB or R*_i_*AB) and KaiC-KaiB-KaiA (R*_i_*BA).

In the phosphorylation phase, KaiC hexamer should be mostly in T*_i_*A states with *i* increases from 0 to 6 as time proceeds due to the kinase activity of KaiA. In the dephosphorylation phase, KaiC hexamer should be mostly in R*_i_*B states with *i* decreases from 6 to 0 as time proceeds due to the effect of KaiB. We assume that the main routes of transition between T and R states are R_0_+A→T_0_A and T_6_+B→R_6_B. As these reactions constitute a closed loop, it may seem trivial for this system to show the oscillatory behavior of KaiC states. However, if we start from the initial condition in which the population of KaiC hexamer is localized at any single state, population rapidly diverges to multiple states by stochastic fluctuation of the timing of each step and eventually the system is dominated by the steady flow of the population.

We circumvent this problem by introducing the following two modes of shuffling: 1) Monomers can be exchanged between two KaiC hexamers in R states as R*_i_*+R*_j_*→R*_k_*+R*_l_* with *i*+*j* = *k*+*l*, and can not be exchanged between two KaiC hexamers in T states. This assumption is consistent with the observation [14] that the shuffling is most frequent during the dephosphorylation phase. 2) Since shuffling is most frequent for the period of several hours in the beginning of the dephosphorylation phase [14], we introduce the additional shuffling reactions involving the R_6_ state, which is populated at this period, and the T state as R_6_+T*_j_*→R*_k_*+R*_l_* with 6+*j* = *k*+*l*. This assumption implies that R state is more accessible than T state for a newly formed hexamer after shuffling. With these assumptions, the oscillatory behavior can be brought about as follows. If the transition from T_6_ to R_6_ is slow compared to the phosphorylation/dephosphorylation, the population of KaiC initially accumulates at the T_6_ state. When the amount of hexamers in the T_6_ state exceeds some threshold, the R_6_ state begins to be populated. Subsequently, this further triggers the autocatalytic increase of KaiC in R states via the shuffling reaction R_6_+T*_j_*→R*_k_*+R*_l_* in a relatively short time. Overall, this autocatalytic allosteric transition acts as a pacemaker which makes the KaiC population coherent enough to maintain the oscillatory behavior. Further details of reactions in Fig. 1 are explained in Model and Methods section and kinetic constants for those reactions are summarized in Supporting Information.

Reactions *in vivo* appear to be essentially same as those reconstituted *in vitro* [14]. Reactions *in vivo*, however, involve only small number of molecules in a single cell: Considering the cyanobactrium cell size of several µm, concentrations of KaiA, KaiB, and KaiC in the reaction mixture in Ref.14 (1.2 µM, 3.5 µM, and 3.5 µM of monomers, respectively) correspond to about 10^4^ to 10^5^ molecules of each Kai monomer in a cell. In order to check whether such smallness in number of molecules affects the reactions, we simulate the stochastic dynamics of reactions in the model of Fig. 1 by using the Gillespie algorithm [26]. The reaction mixture confined in a µm vessel is simulated with 10000 dimers of KaiA, 30000 dimers of KaiB, and 10000 hexamers of KaiC, which corresponds to 1:3:3 ratio of monomers. Since the oscillation should be less stable in a more noisy system with smaller numbers of molecules, the stable persistent oscillation of reactions observed in this stochastic simulation will assure both the stability of oscillation in a cell of µm size and the stability of oscillation *in vitro* with the macroscopic number of molecules.

## Results

**Figure 2:**
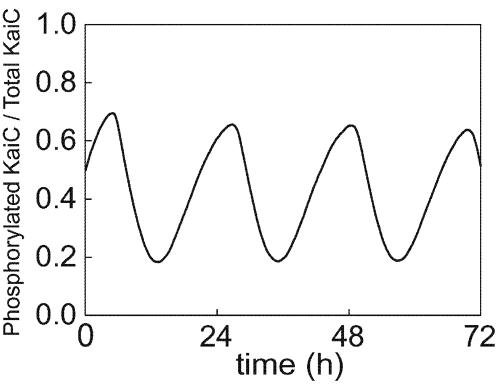
The simulated oscillation in the phosphorylation level *p*(*t*).

**Figure 3:**
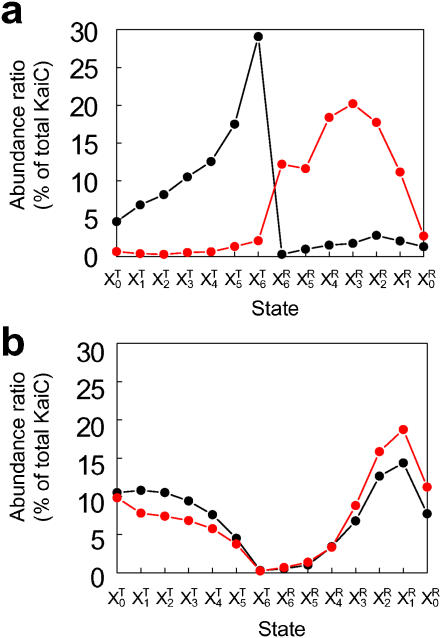
**Typical examples of distributions of KaiC states,** ***P*** **(R** ***_i_,t*** **) =** ***X_i_*** **^R^**/***X*** **and** ***P*** **(T** ***_i_*** **,** ***t*** **) =** ***X_i_*** **^T^/** ***X*** **.** (a) Distributions at *t* = 18 hours (black line) and *t* = 22 hours (red line) calculated with the standard parameter set consistent with the autocatalytic increase of KaiC population in R states. (b) Distributions at *t* = 10 hours (black line) and *t* = 14 hours (red line) calculated with the transition rate from T_6_ to R_6_ increased 100-fold from its standard value.

As shown in Fig. 2, the model exhibits a stable persistent oscillation in the level of KaiC phosphorylation, *p*(*t*) = Σ*_i_ i*(*X_i_* ^R^+*X_i_* ^T^)/6*X*, where *X* = ) = Σ*_i_* (*X_i_* ^R^+*X_i_* ^T^), *X_i_* ^R^ = [R*_i_*]+[R*_i_*A]+[R*_i_*B]+[R*_i_*AB]+[R*_i_*BA], *X_i_* ^T^ = [T*_i_*]+[T*_i_*A]+[T*_i_*AB], and […] denotes the concentration of each of the chemical species. With the parameters used (summarized in Supplementary Table S1), the phosphorylation level of KaiC oscillates between 0.2 and 0.8 and the period length is about 22 hours. These findings are consistent with the experimental observations (Nakajima et al Science, Kageyama et al Mol Cell). This oscillatory behavior is necessarily accompanied by the autocatalytic increase of KaiC population in R states described above. To illustrate how the mechanism works, typical examples of distributions of KaiC states at time *t*, *P*(R*_i_*,*t*) = *X_i_* ^R^/*X* and *P*(T*_i_*,*t*) = *X_i_* ^T^/*X*, are plotted in Fig. 3. When the rate of transition from T_6_ to R_6_ is 100 times faster than its standard value, KaiC hexamers are not caught at the T_6_ state and leak to the R_6_ state, which promotes active monomer shuffling between T and R. Through this active shuffling the population spreads over the R states, which suppresses the oscillation as shown in Fig. 3b. The oscillation is also suppressed when the shuffling rates are low. With the lower shuffling rates, the small amount of KaiC hexamers appeared at the R_6_ state do not trigger further increase at the R states via reactions R_6_+T*_j_*→R*_k_*+R*_l_* (data not shown).

**Figure 4:**
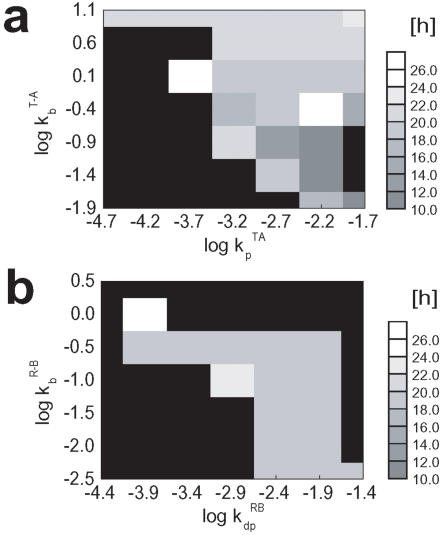
The parameter dependence of the period length of oscillation in the phosphorylation level *p*(*t*). The period length is shown in gray scale against (a) log*k_p_* ^TA^ and log*k_b_* ^T-A^ or (b) log*k_dp_* ^RB^ and log*k_b_* ^R-B^. The oscillation dies out in the black area.

The model also predicts the dependence of the oscillation on various rate constants. For example, *k_p_* ^TA^[T*_i_*A], which is the rate of phosphorylation from T*_i_*A to T*_i_*_+1_A, should affect the period length of oscillation, and [T*_i_*A] is strongly dependent on the binding rate *k_b_* ^T-A^ of A to T*_i_*. (See Model and Methods for the precise definition of these constants.) In Fig. 4a, the oscillation period length is plotted against *k_p_* ^TA^ and *k_b_* ^T-A^. The persistent oscillation exists in the parameter region of *k_p_* ^TA^ *k_b_* ^T-A^ ∼8.6×10^−5^sec^−2^, and in the region of *k_b_* ^T-A^∼5 sec^−1^. The oscillation should also depend on *k_dp_* ^RB^[R*_i_*_+1_B], which is the rate of dephosphorylation from R*_i_*_+1_B to R*_i_*B. In Fig. 4b, the oscillation period is plotted against *k_dp_* ^RB^ and *k_b_* ^R-B^, where *k_b_* ^R-B^ is the rate constant of binding of B to R*_i_*. The stable oscillation is found in the parameter region of *k_dp_* ^RB^ *k_b_* ^R-B^∼1.1×10^−4^ sec^−2^. In wide areas of Figs. 4a and 4b, the oscillation period length does not vary much as far as the parameters are consistent with the autocatalytic increase of KaiC population in R states. The period varies, however, on the line of *k_p_* ^TA^ *k_b_* ^T-A^∼8.6×10^−5^ sec^−2^. and the period length tends to be extended when the parameters are close to the boundary of the stable oscillation.

The oscillation can be further characterized by examining the association dynamics among Kai proteins. In the following, the simulated association dynamics are compared with the experimental results of Ref.14 from several aspects.

### Dependence on concentrations of Kai proteins

**Figure 5 pone-0000408-g005:**
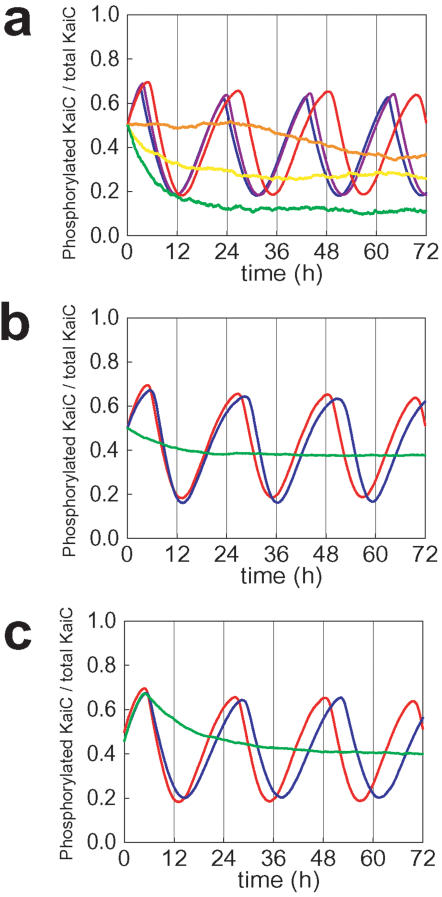
Concentration dependence of the simulated time course of KaiC phosphorylation level, *p*(*t*). (a) *p*(*t*) for the standard mixture (red) and *p*(*t*) calculated with the concentrations of KaiA, KaiB, and KaiC increased 2.5-fold (blue), 5-fold (purple) or decreased to 1/10 (orange), 1/20 (yellow), and 1/40 (green) of their standard values. (b) *p*(*t*) calculated with the KaiA concentration decreased to 2/3 (blue) or 1/4 (green) of its standard value (red). (c) *p*(*t*) calculated with the KaiB concentration decreased to 2/3 (blue) or 1/3 (green) of its standard value (red).

It was experimentally observed that the oscillation is persistent when concentrations of Kai proteins are increased from their standard values, but the oscillation dies out when the concentrations are decreased to 1/10 or less [14]. When the concentration of KaiC is fixed, the oscillation is stable against a small change in concentration of KaiA or KaiB, but the oscillation dies out when the KaiA concentration is decreased to 1/4 or the KaiB concentration is decreased to 1/3 of their standard values [14]. These features are well reproduced in the simulation as shown in Fig. 5. In the model, these features are largely explained by the Michaelis-Menten kinetics of binding among Kai proteins. With the standard values of concentrations of Kai proteins, the binding reaction rates are saturated and insensitive to the small changes in concentrations of Kai proteins. With the decreased concentration, however, the binding rates leave this saturated regime and begin to sensitively depend on the concentrations, which disturbs the balance among the reaction rates required for the oscillation.

### Kai protein complex formation

**Figure 6 pone-0000408-g006:**
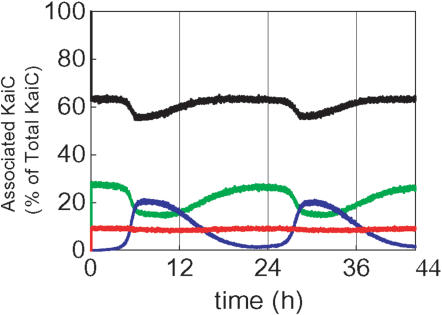
Dynamics of protein association as shown by the amount of the KaiC-KaiA complex (*P*_AC_, green), the KaiC-KaiB complex (*P*_BC_, blue), the KaiC-KaiA-KaiB and KaiC-KaiB-KaiA complexes (*P*_ABC_, red), and the free KaiC (*P*_free_, black). The amounts are expressed as percentage of the total number of KaiC hexamers.

We refer to the probability for a KaiC hexamer to be in complex with KaiA or with KaiB as *P*_AC_ and *P*_BC_, respectively, and the probability for a KaiC to be in complex with KaiA and KaiB as *P*_ABC._ Then, the ratio of the free KaiC hexamer is expressed as *P*_free_ = 1−(*P*_AC_+*P*_BC_+*P*_ABC_). In Ref.14, it was experimentally observed that *P*_free_ oscillates between 55–80% and *P*_BC_ oscillates between 7–18%, which are 180° out of phase with each other. *P*_AC_ oscillates between 8–12% in phase with that of *P*_free_, and *P*_ABC_ oscillates between 10–14% in phase close to that of *P*_BC_. Temporal changes of corresponding values in our simulation are plotted in Fig. 6, which are obtained by calculating *P*_AC_ = Σ*_i_*([T*_i_*A]+[R*_i_*A])/*X, P*_BC_ = Σ*_i_*[R*_i_*B]/*X*, and *P*_ABC_ = Σ*_i_*([T*_i_*AB]+[R*_i_*AB]+[R*_i_*BA])/*X*. The overall features of the experiment are mostly reproduced in Fig. 6: The simulated oscillations of *P*_free_ and *P*_BC_ resemble the experimental data, and *P*_AC_ oscillates with the same oscillation phase as was observed in the experiment. The range of oscillation of *P*_AC_, however, is 18–30% in the model, which is consistently higher than the experimentally observed range of 8–12%, and *P*_ABC _oscillates only weakly in Fig. 6. Such disagreements may be due to the inappropriate description of binding of B to AC in the model. Further experimental information on the binding mode of KaiB to KaiC-KaiA complex is desired.

### Kinetics of KaiC-KaiA and KaiC-KaiB association

**Figure 7 pone-0000408-g007:**
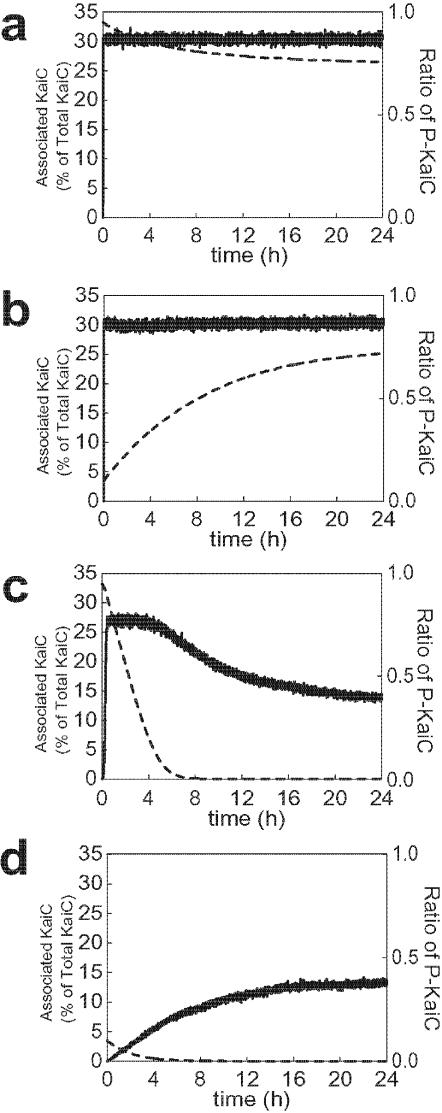
Interplay of the KaiC-KaiA and KaiC-KaiB association and the phosphorylation level of KaiC. The amount of associated KaiC is shown in solid lines as percentage of the total number of KaiC hexamers, whereas the phosphorylation level, *p*(*t*), is shown in dashed lines. (a) KaiC with *p*(0) ∼95% is incubated with KaiA, (b) KaiC with *p*(0) ∼5% is incubated with KaiA, (c) KaiC with *p*(0) ∼95% is incubated with KaiB, and (d) KaiC with *p*(0) ∼5% is incubated with KaiB.

In the experiment, when KaiC with the phosphorylation level ∼95% was incubated in the presence of KaiA, the phosphorylation level was kept constant at ∼95% and the KaiC-KaiA complex was readily formed to give *P*_AC_ ∼10% [14]. On the other hand, when KaiC with the phosphorylation level ∼5% was incubated in the presence of KaiA, it took ∼6 hours for the phosphorylation level to increase to ∼95%, while the KaiC-KaiA complex was readily formed to make *P*_AC_ ∼10% from the beginning [14]. These observations indicate that the binding affinity of KaiA to KaiC does not depend on the phosphorylation level and the rate of KaiC-KaiA binding is much faster than that of phosphorylation. As shown in Figs. 7a and 7b, these features are qualitatively reproduced by our model: Starting from the solution of KaiA and KaiC with *p*(0) ∼95%, *p*(*t*) approaches ∼75% in the model and *P*_AC_ stays at ∼30%. Starting from the solution of KaiA and KaiC with *p*(0) ∼5%, it took 12 hours for *p*(*t*) to increase to ∼75%, while *P*_AC_ stays at ∼30% from the beginning.

Kinetics of KaiC-KaiB association was also measured experimentally [14]. When KaiC with the phosphorylation level ∼95% was incubated with KaiB, it took 8 hours for the phosphorylation level to decrease to 5% and the phosphorylation level further decreased to less than 5%. *P*_BC_ increased to 20% within the first 8 hours and then gradually decreased to 10%. When KaiC with the phosphorylation level ∼5% was incubated with KaiB, on the other hand, the phosphorylation level decreased to ∼0%, while *P*_BC_ increased to 10% [14]. Thus, KaiB binds strongly to highly phosphorylated KaiC but only weakly to KaiC with the phosphorylation level<5%. Most of these features are qualitatively reproduced by the model as shown in Figs. 7c and 7d: As the phosphorylation level decreases, KaiC accumulates in the T_0_ state, which in turn decreases the binding affinity of KaiB to KaiC.

### Shuffling of KaiC monomers

**Figure 8:**
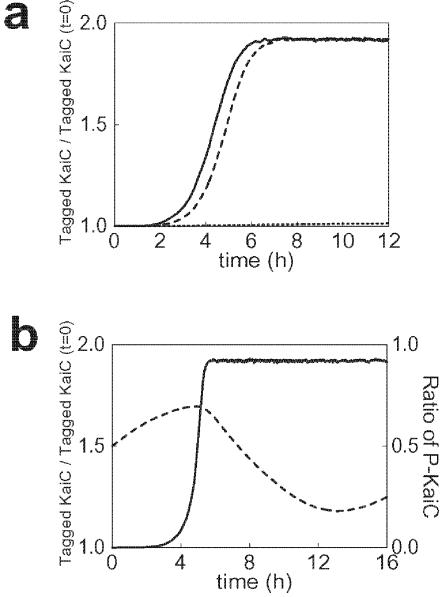
Monomer shuffling between KaiC hexamers. (a) KaiC hexamers composed of tagged monomers and those composed of untagged monomers were mixed in the ratio of 1:1 in the absence of KaiA or KaiB (real line), in the presence of KaiB (dashed line), or in the absence of KaiA (dotted line). The amounts of tagged KaiC hexamers normalized by its initial amount are shown. (b) The phosphorylation level *p*(*t*) (dashed line) and the amount of tagged KaiC hexamers normalized by its initial amount (solid line) in the presence of KaiA and KaiB.

It has been shown experimentally that two KaiC hexamers can interact with each other and exchange monomers between them [14]. After the KaiC hexamers composed of tagged monomers and those without a tagged monomer were mixed at a ratio of 1:1, the kinetics of shuffling was followed by the increase of tagged KaiC hexamers. In the absence of KaiA or KaiB or in the presence of KaiB, the number of tagged KaiC hexamers became twice of the initial amount in about 4 hours, showing that all the KaiC hexamers interact and exchange monomers within this time interval. In the presence of KaiA, however, the amount of tagged KaiC monomers did not increase as much as that in the above cases. In the presence of KaiA and KaiB, the shuffling activity was low at the beginning but rose rapidly after the phosphorylation level had reached a maximum [14]. These features are also qualitatively reproduced in the model as shown in Fig. 8.

### Temperature compensation

**Figure 9:**
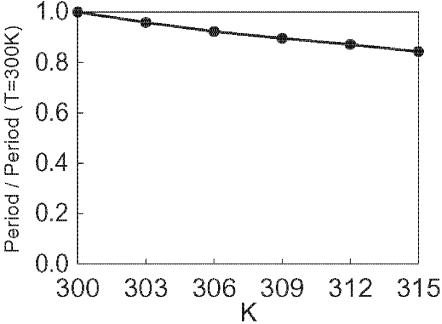
Temperature compensation of the oscillation period length of the phosphorylation level *p*(*t*). The period length is normalized by its value at 300K.

It was observed that, with the change of temperature from 25°C to 35°C, the oscillation period length was shortened only by 5% (from 22 h to 21 h) [7]. The model provides a possible explanation of this weak temperature dependence. As shown in Fig. 1, the model has two opposite pathways of phosphorylation and dephosphorylation within the both T and R states. In T state, for example, when the dephosphorylation process has higher activation energy than the phosphorylation process, the dephosphorylation rate approaches the phosphorylation rate at an elevated temperature. This effect should counteract the acceleration of reactions due to the temperature increase in both T and R states, yielding the weaker temperature dependence of the oscillation period. The simulation results of Fig. 9 show that the period is indeed temperature-compensated through this mechanism, so that the period is shortened only by 10% in the temperature increase from 300K to 309K, even though time constant of each reaction decreases by 26–32%. See Model and Methods for the temperature dependence of individual reactions defined in the model.

## Discussion

In this paper, we constructed a model of oscillation of KaiC phosphorylation and showed that the model can qualitatively reproduce the hitherto published experimental data. However, this model has some quantitative disagreements with the experimental data, such as the oscillation range of *P*_AC_ in Fig. 6 or the phosphorylation level in Fig. 7. Although some of these points may be remedied by introducing further reaction channels in the model, such revision of the model would not strengthen the plausibility of the model at this point with the lack of the corresponding experimental information. Instead, we here demonstrated that a simple model is enough to capture the essential features of the oscillation observed in Ref. 7 and those of the association dynamics among Kai proteins observed in Ref. 14.

The model, however, still has a room for improvement. Possible extensions include the introduction of the process of ATP hydrolysis and phosphorylation at two phosphorylation sites, Ser431 and Thr432. Since the ATP binding site and two phosphorylation sites are close in space in the crystal structure of KaiC hexamer [25], the timing of phosphorylation or dephosphorylation at two sites should be correlated with each other, as well as with the timing of hydrolysis, capture, or release of the nucleotide. This correlation may well have significant effect on the stability and robustness of the the autocatalytic increase of KaiC population in R states discussed in this paper.

The present model is based on the two main assumptions, the shuffling of KaiC monomers, and the allosteric transition between R and T states of KaiC hexamer. It should be noted that both of these assumptions are natural consequences from the experimental observations. Overall qualitative agreement between the present model and the experimental data suggests that the monomer shuffling and the allosteric transition constitute a sufficient condition for the circadian oscillation of KaiC phosphorylation. Furthermore, the analysis on the population dynamics provides an explanation on why KaiC hexamers can synchronize in their phosphorylation level: the autocatalytic increase of KaiC population in R states can adjust the otherwise incoherent levels of phosphorylation. Comparison of the further improved model and the detailed experiments will provide a deeper understanding of a core mechanism of circadian rhythmicity.

## Methods

### Model and Methods

In this section, the kinetic scheme of each reaction in the model is explained.

#### Autokinase/phosphatase activity

In the absence of KaiA or KaiB, the phosphorylation level is low at equilibrium at room temperature, showing that the weak autophosphatase activity of KaiC is dominant [14], [16], [27] over the even weaker autokinase activity [28]. Thus, we assume that KaiC is dephosphorylated with the rate *k_dp_* ^T^[T*_i_*_+1_] or *k_dp_* ^R^[R*_i_*_+1_] for *i* = 0 to 5. When complexed with KaiA, KaiC is phosphorylated [14] with the rate *k_p_* ^TA^[T*_i_*A] for *i* = 0 to 5 or *k_p_* ^RA^[R*_i_*A] for *i* = 1 to 5. When complexed with KaiB, KaiC in the R state is dephosphorylated with the rate *k_dp_* ^RB^[R*_i_*_+1_B] for *i* = 0 to 5. We require that the catalyzed dephosphorylation should be faster than the autodephosphorylation; *k_dp_* ^RB^>*k_dp_* ^T^≈*k_dp_* ^R^. Reactions in the KaiABC complex have not yet been specified experimentally, but we assume that KaiC in T*_i_*AB or R*_i_*AB is phosphorylated as in T*_i_*A or R*_i_*A with the rate *k_p_* ^TAB^[T*_i_*AB] for *i* = 0 to 5 or *k_p_* ^RAB^[R*_i_*AB] for *i* = 1 to 5, and that KaiC in R*_i_*BA is dephosphorylated as in R*_i_*B with the rate *k_dp_* ^RBA^[R*_i_*_+1_BA] for *i* = 0 to 5. If we assume equal phosphorylation rates for all unphosphorylated monomers, the phosphorylation rate for hexamer decreases as phosphorylation proceeds. Likewise, if we assume equal dephosphorylation rates for all phosphorylated monomers, the dephosphorylation rate for hexamer decreases as dephosphorylation proceeds. This assumption, however, centralize KaiC hexamer population around the states with *i* = 3 to 4, which suppresses the oscillation. Instead, we here assume that the reaction takes place at a specific monomer in each KaiC hexameric state, so that *k_dp_* ^T^, *k_dp_* ^R^, *k_p_* ^TA^, *k_p_* ^RA^, *k_dp_* ^RB^, *k_p_* ^TAB^, *k_p_* ^RAB^, and *k_dp_* ^RBA^ are assumed to be independent of *i*.

#### Association/dissociation of Kai proteins

Homooligomers of KaiC (T*_i_* or R*_i_*), and KaiA in solution are assumed to be a hexamer [14], [22], [23] and a dimer [14], [27], [29]–[31], respectively. KaiA is supposed to be dimeric when bound to KaiC hexamer [14], [32]. We assume that KaiA binds to KaiC with the rate *k_b_* ^T-A^[A][T*_i_*]/(*K_M_* ^T-A^+[*A*]) or *k_b_* ^R-A^[A][R*_i_*]/(*K_M_* ^R-A^+[*A*]), where [A] is the concentration of KaiA dimer. This rather ad hoc assumption of Michaelis-Menten kinetics is required for explaining the invariability of the period length over the broad range of concentration of Kai proteins observed experimentally (Fig. 5). This suggests the possibility that the formation of KaiC-KaiA complex is preceded by the weak encounter complex which is in equilibrium with the uncomplexed KaiA and KaiC. KaiA dissociates from the KaiC-KaiA complex with the rate *k_d_* ^TA^[T*_i_*A] or *k_d_* ^RA^[R*_i_*A]. We assume that R_0_A is rapidly brought into T_0_A as will be explained later and we omit the KaiA dissociation from R_0_A. As the binding kinetics between KaiA and KaiC does not depend on the phosphorylation level of KaiC [14], we assume that the constants *k_b_* ^T-A^, *k_b_* ^R-A^, *K_M_* ^T-A^, *K_M_* ^R-A^, *k_d_* ^TA^, and *k_d_* ^RA^ are independent of *i*.

KaiB is assumed to be a dimer in solution [14], [17], [30], and is supposed to be tetrameric when bound to KaiC, since the tetrameric form of KaiB was observed to be functionally important [33], [34]. Concentration of KaiB tetramer is proportional to [B]^2^, where [B] is the concentration of KaiB dimer, so that KaiB binds to KaiC with the rate *k_b_* ^R-B^[B]^2^[R*_i_*]/(*K_M_* ^R-B^+[B]^2^) or 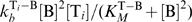. The binding rate of KaiB is large when KaiC is phosphorylated [14], so that we simply put 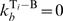 for *i* = 0 to 5 and 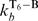 to be nonzero. KaiB dissociates from R*_i_*B with the rate *k_d_* ^RB^[R*_i_*B]. We assume that T_6_B is rapidly brought into R_6_B as will be explained later and we omit the KaiB dissociation from T_6_B. For simplicity we assume that *k_b_* ^R-B^, *K_M_* ^R-B^ and *k_d_* ^RB^are independent of *i*.

Likewise, the KaiC-KaiA-KaiB complex is formed by binding of KaiB to KaiC-KaiA and the KaiC-KaiB-KaiA complex is formed by binding of KaiA to KaiC-KaiB. Even though these two complexes are experimentally indistinguishable at present, we take them into account separately because it is proposed that KaiA and KaiB compete with each other for a common binding site on KaiC hexamer [28]. The binding reactions have the rate *k_b_* ^TA-B^[B]^2^[T*_i_*A]/(*K_M_* ^TA-B^+[B]^2^), *k_b_* ^RA-B^[B]^2^[R*_i_*A]/(*K_M_* ^RA-B^+[B]^2^) or *k_b_* ^RB-A^[A][R*_i_*B]/(*K_M_* ^RB-A^+[A]). KaiA dissociates from the KaiC-KaiB-KaiA complex with the rate *k_d_* ^RBA^[R*_i_*BA] and two KaiB dimers dissociate from the KaiC-KaiA-KaiB complex with the rate *k_d_* ^TAB^[T*_i_*AB] or *k_d_* ^RAB^[R*_i_*AB]. We assume that the constants, *k_b_* ^TA-B^, *k_b_* ^RA-B^, *k_b_* ^RB-A^, *K*_M_^TA-B^, *K*_M_^RA-B^, *K*_M_^RB-A^, *k_d_* ^RBA^, *k_d_* ^TAB^, and *k_d_* ^RAB^ are independent of *i*.

#### Allosteric transitions and shuffling

Conformational transition between the T state and the R state of KaiC hexamer is limited to the KaiC with *i* = 0 or 6, and other possible transitions are neglected. Allosteric transition between T_0_ state and R_0_ state occurs in three reactions: the dominant and catalyzed reaction R_0_+A→T_0_A with the rate 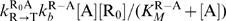, the uncatalyzed reaction R_0_→T_0_ with the rate 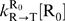, and the uncatalyzed reverse reaction T_0_→R_0_ with the rate 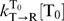. Here, the rates of the uncatalyzed reactions are assumed to be similar and lower than that of the catalyzed reactions: 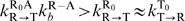. Allosteric transition between T_6_ state and R_6_ state occurs in only one reaction T_6_+B→R_6_B with the rate 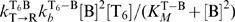. As discussed above, 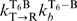 has to be low enough for the the autocatalytic increase of KaiC population in R states to occur so that 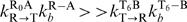. We assume that R_0_A and T_6_B are the special states primed for conformational transition so that phosphorylation or dephosphorylation does not occur from R_0_A and T_6_B.

We assume that monomer shuffling occurs between two KaiC hexamers when both of them are in the R state as R*_i_*+R*_j_*→R*_k_*+R*_l_* with the rate *k_s_* ^RR^[R*_i_*][R*_j_*]. Since shuffling is most frequent for several hours at the beginning of the dephosphorylation phase [14] we introduce the additional shuffling reactions involving the R_6_ state, which is populated at this interval, and the T state as R_6_+R*_j_*→R*_k_*+R*_l_* with the rate *k_s_* ^RT^[R_6_][T*_j_*]. Note that the resulting two monomers are again assumed to be both in the R state. This amounts to an assumption that the R state is entropically stabilized and is more easily formed than the T state which takes more time to be realized after shuffling. For both types of shuffling reactions, we assume that monomers are randomly exchanged between two hexamers, which results in the binomial distribution of *k* and *l* with *i*+*j* = *k*+*l*. For simplicity, we assume *k_s_* ^RR^ and *k_s_* ^RT^do not depend on *i* or *j*.

### Temperature dependence

We assume that the rate constants defined above depend on temperature as *k*∝exp(−Δ*E*/*k*_B_ *T*), where *k*_B_ is the Boltzmann constant. Though there is no available experimental data to estimate Δ*E* for each reaction, it would be reasonable to assume that Δ*E* should be within the range of typical biochemical processes of 10-20*k*_B_ *T*. For most of the rate constants the activation energy was set to be Δ*E* = 10*k*_B_ *T*_0_ with *T*_0_ = 300K, but 12*k*_B_ *T*_0_ for *k_d_* ^TA^, *k_d_* ^TAB^, *k_b_* ^R-A^, *k_d_* ^RB^, *k_d_* ^RAB^, *k_b_* ^RA-B^ and 13*k*_B_ *T*_0_ for *k_dp_* ^T^, *k_p_* ^RA^, and *k_p_* ^RAB^.

### Parameters and initial conditions

Values of parameters are summarized in Supplementary Table S1. The oscillatory behavior in the model does not sensitively depend on the parameter values. As there is no direct experimental observation to determine these values, they were manually tuned to fit the experimental data semi-quantitatively in Figs. 4–8. When the reaction mixture was prepared by mixing recombinant Kai proteins, KaiC seemed to be phosphorylated to some extent [14]. We therefore start our simulations from the initial condition that KaiC is populated at the T state with the distribution of phosphorylation level *i* to be *p_i_* = 6!/(*i*!(6−*i*)!)0.5*^i^*0.5^6−*i*^ except for the simulations in Fig. 7. In Figs. 7a and c the initial distribution is *p_i_* = 6!/(*i*!(6−*i*)!)0.95*^i^*0.05^6−*i*^ and in Figs. 7b and d *p_i_* = 6!/(*i*!(6−*i*)!)0.05*^i^*0.095^6−*i*^.

## Supporting Information

Table S1 (0.31 MB DOC)
