# Supplementary material for: Monomer-Shuffling and Allosteric Transition in KaiC Circadian Oscillation
Source: PLoS One. 2007 May 2;2(5):e408. doi: 10.1371/journal.pone.0000408 (PMC1853237; doi:10.1371/journal.pone.0000408)
Supplement: Table S1 — (0.31 MB DOC) [file pone.0000408.s001.doc]

**Supplementary Table**: Parameter values

| Reaction type | Reaction scheme | Rate  constants | Values as used in the simulation | *Values in standard units |
| --- | --- | --- | --- | --- |
| Phosphorylation | $\text{T}_{i}\text{A}\to\text{T}_{i+1}\text{A}$ | $k_{p}^{\text{TA}}$ | $1.3\times10^{-2} [\Delta t^{-1}]$ | $6.1\times10^{-4} [\sec^{-1}]$ |
|  | $\text{T}_{i}\text{AB}\to\text{T}_{i+1}\text{AB}$ | $k_{p}^{\text{TAB}}$ | $1.3\times10^{-2} [\Delta t^{-1}]$ | $6.1\times10^{-4} [\sec^{-1}]$ |
|  | $\text{R}_{i}\text{A}\to\text{R}_{i+1}\text{A}$ | $k_{p}^{\text{RA}}$ | $1.75\times10^{-2} [\Delta t^{-1}]$ | $8.2\times10^{-4} [\sec^{-1}]$ |
|  | $\text{R}_{i}\text{AB}\to\text{R}_{i+1}\text{AB}$ | $k_{p}^{\text{RAB}}$ | $1.75\times10^{-2} [\Delta t^{-1}]$ | $8.2\times10^{-4} [\sec^{-1}]$ |
| Dephosphorylation | $\text{T}_{i}\to\text{T}_{i-1}$ | $k_{dp}^{\text{T}}$ | $3.7\times10^{-3} [\Delta t^{-1}]$ | $1.7\times10^{-4} [\sec^{-1}]$ |
|  | $\text{R}_{i}\to\text{R}_{i-1}$ | $k_{dp}^{\text{R}}$ | $1.0\times10^{-3} [\Delta t^{-1}]$ | $4.7\times10^{-5} [\sec^{-1}]$ |
|  | $\text{RB}_{i}\to\text{RB}_{i-1}$ | $k_{dp}^{\text{RB}}$ | $2.5\times10^{-2} [\Delta t^{-1}]$ | $1.2\times10^{-3} [\sec^{-1}]$ |
|  | $\text{RBA}_{i}\to\text{RBA}_{i-1}$ | $k_{dp}^{\text{RBA}}$ | $2.5\times10^{-2} [\Delta t^{-1}]$ | $1.2\times10^{-3} [\sec^{-1}]$ |
| Association | $\text{T}_{i}\to\text{T}_{i}\text{A}$ | $k_{b}^{\text{T-A}}$ | $3.0 [\Delta t^{-1}]$ | $1.4\times10^{-1} [\sec^{-1}]$ |
|  |  | $K_{M}^{\text{T-A}}$ | $1.0\times10^{3}$ | $7.5\times10^{-8} [\text{M}]$ |
|  | $\text{T}_{i}\text{A}\to\text{T}_{i}\text{AB}$ | $k_{b}^{\text{TA-B}}$ | $1.0 [\Delta t^{-1}]$ | $4.7\times10^{-2} [\sec^{-1}]$ |
|  |  | $K_{M}^{\text{TA-B}}$ | $2.5\times10^{4}$ | $1.4\times10^{-16} [\text{M}^{2}]$ |
|  | $\text{R}_{i}\to\text{R}_{i}\text{A}$ | $k_{b}^{\text{R-A}}$ | $2.0 [\Delta t^{-1}]$ | $9.4\times10^{-2} [\sec^{-1}]$ |
|  |  | $K_{M}^{\text{R-A}}$ | $3.0\times10^{3}$ | $2.3\times10^{-7} [\text{M}]$ |

| Reaction type | Reaction scheme | Rate  constants | Values as used in the simulation | *Values in standard units |
| --- | --- | --- | --- | --- |
| Association | $\text{R}_{i}\text{A}\to\text{R}_{i}\text{AB}$ | $k_{b}^{\text{RA-B}}$ | $0.5 [\Delta t^{-1}]$ | $2.3\times10^{-2} [\sec^{-1}]$ |
|  |  | $K_{M}^{\text{RA-B}}$ | $2.5\times10^{4}$ | $1.4\times10^{-16} [\text{M}^{2}\text{]}$ |
|  | $\text{R}_{i}\to\text{R}_{i}\text{B}$ | $k_{b}^{\text{R-B}}$ | $2.0 [\Delta t^{-1}]$ | $9.4\times10^{-2} [\sec^{-1}]$ |
|  |  | $K_{M}^{\text{R-B}}$ | $5.0\times10^{7}$ | $2.8\times10^{-13} [\text{M}^{2}\text{]}$ |
|  | $\text{R}_{i}\text{B}\to\text{R}_{i}\text{BA}$ | $k_{b}^{\text{RB-A}}$ | $0.5 [\Delta t^{-1}]$ | $2.3\times10^{-2} [\sec^{-1}]$ |
|  |  | $K_{M}^{\text{RB-A}}$ | $5.0\times10^{2}$ | $2.9\times10^{-8} [\text{M}]$ |
| Dissociation | $\mathrm{T}_{i}\text{A}\to\text{T}_{i}$ | $k_{d}^{\text{TA}}$ | $6.0$ | $2.8\times10^{-1} [\sec^{-1}]$ |
|  | $\text{T}_{i}\text{AB}\to\text{T}_{i}\text{A}$ | $k_{d}^{\text{TAB}}$ | $3.0$ | $1.4\times10^{-1} [\sec^{-1}]$ |
|  | $\text{R}_{i}\text{A}\to\text{R}_{i}$ | $k_{d}^{\text{RA}}$ | $5.0$ | $2.3\times10^{-1} [\sec^{-1}]$ |
|  | $\text{R}_{i}\text{AB}\to\text{R}_{i}\text{A}$ | $k_{d}^{\text{RAB}}$ | $2.0$ | $9.4\times10^{-2} [\sec^{-1}]$ |
|  | $\text{R}_{i}\text{B}\to\text{R}_{i}$ | $k_{d}^{\text{RB}}$ | $5.0$ | $2.3\times10^{-1} [\sec^{-1}]$ |
|  | $\text{R}_{i}\text{BA}\to\text{R}_{i}\text{B}$ | $k_{d}^{\text{RBA}}$ | $2.0$ | $9.4\times10^{-2} [\sec^{-1}]$ |

| Reaction type | Reaction scheme | Rate  constants | Values as used in the simulation | *Values in standard units |
| --- | --- | --- | --- | --- |
| Allosteric  transition | $\text{T}_{6}\to\text{R}_{6}\text{B}$ | $k_{\text{T}\to\text{R}}^{\text{T}_{6}\text{B}}k_{b}^{\text{T}_{6}\text{-B}}$ | $1.0\times10^{-4}$ | $4.7\times10^{-6} [\sec^{-1}]$ |
|  |  | $K_{M}^{\text{T-B}}$ | $5.0\times10^{4}$ | $2.8\times10^{-16} [\text{M}]$ |
|  | $\text{R}_{0}\to\text{T}_{0}\text{A}$ | $k_{\text{R}\to\text{T}}^{\text{R}_{0}\text{A}}k_{b}^{\text{R-A}}$ | $5.0\times10^{-3}$ | $2.3\times10^{-4} [\sec^{-1}]$ |
|  |  | $K_{M}^{\text{R-A}}$ | $5.0\times10^{2}$ | $3.8\times10^{-8} [\text{M}]$ |
|  | $\text{R}_{0}\to\text{T}_{0}$ | $k_{\text{R}\to\text{T}}^{\text{R}_{0}}$ | $7.0\times10^{-4}$ | $3.3\times10^{-5} [\sec^{-1}]$ |
|  | $\text{T}_{0}\to\text{R}_{0}$ | $k_{\text{T}\to\text{R}}^{\text{T}_{0}}$ | $5.0\times10^{-4}$ | $2.3\times10^{-5} [\sec^{-1}]$ |
| Shuffling | $\text{R}_{i}+\text{R}_{j}\to\text{R}_{k}+\text{R}_{l}$ | $k_{s}^{\text{RR}}$ | $1.0\times10^{-4}$ | $3.0\times10^{-4} [\text{M}^{-1}\cdot\sec^{-1}]$ |
|  | $\text{R}_{6}+\text{T}_{i}\to\text{R}_{k}+\text{R}_{l}$ | $k_{s}^{\text{RT}}$ | $3.0\times10^{-5}$ | $9.0\times10^{-5} [\text{M}^{-1}\cdot\sec^{-1}]$ |

*Values as used in the simulation are converted to those in standard units by assuming the time unit in the simulation, Δ*t* = 21.3sec, and the volume of a cell to be $22.1\times10^{-15}$*l*.
